# *N*-(2-Allyl-4-eth­oxy-2*H*-indazol-5-yl)-4-methyl­benzene­sulfonamide

**DOI:** 10.1107/S1600536814009283

**Published:** 2014-04-30

**Authors:** Hakima Chicha, El Mostapha Rakib, Latifa Bouissane, Maurizio Viale, Mohamed Saadi, Lahcen El Ammari

**Affiliations:** a Laboratoire de Chimie Organique et Analytique, Université Sultan Moulay Slimane, Faculté des Sciences et Techniques, Béni-Mellal, BP 523, Morocco; b IST Istituto Nazionale per la Ricerca sul Cancro, U.O.C. Terapia Immunologica, L. go R. Benzi 10, 16132 Genova, Italy; c Laboratoire de Chimie du Solide Appliquée, Faculté des Sciences, Université Mohammed V-Agdal, Avenue Ibn Battouta, BP 1014, Rabat, Morocco

## Abstract

The indazole ring system of the title compound, C_19_H_21_N_3_O_3_S, is almost planar (r.m.s. deviation = 0.0192 Å) and forms dihedral angles of 77.99 (15) and 83.9 (3)° with the benzene ring and allyl group, respectively. In the crystal, centrosymmetrically related mol­ecules are connected by pairs of N—H⋯O hydrogen bonds into dimers, which are further linked by C—H⋯O hydrogen bonds, forming columns parallel to the *b* axis.

## Related literature   

For the biological activity of sulfonamides, see: Drews (2000 ▶); Supuran & Scozzafava (2001 ▶); Abbate *et al.* (2004 ▶); Rostom (2006 ▶); Ghorab *et al.* (2009 ▶). For similar compounds, see: Bouissane *et al.* (2006 ▶); Abbassi *et al.* (2012 ▶, 2013 ▶).
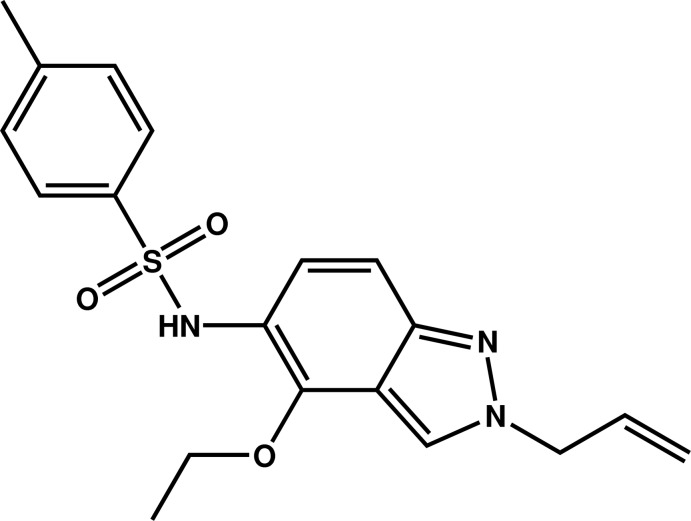


## Experimental   

### 

#### Crystal data  

C_19_H_21_N_3_O_3_S
*M* *_r_* = 371.45
Monoclinic, 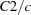

*a* = 26.0808 (5) Å
*b* = 7.9335 (2) Å
*c* = 21.1573 (4) Å
β = 122.839 (1)°
*V* = 3678.13 (14) Å^3^
*Z* = 8
Mo *K*α radiation
μ = 0.20 mm^−1^
*T* = 296 K
0.42 × 0.35 × 0.30 mm

#### Data collection  

Bruker X8 APEX diffractometer
Absorption correction: multi-scan (*SADABS*; Bruker, 2009 ▶) *T*_min_ = 0.693, *T*_max_ = 0.747
37135 measured reflections
4059 independent reflections
3100 reflections with *I* > 2σ(*I*)
*R*_int_ = 0.048

#### Refinement  

*R*[*F* ^2^ > 2σ(*F* ^2^)] = 0.046
*wR*(*F* ^2^) = 0.134
*S* = 1.07
4059 reflections
235 parameters
H-atom parameters constrained
Δρ_max_ = 0.28 e Å^−3^
Δρ_min_ = −0.36 e Å^−3^

### 

Data collection: *APEX2* (Bruker, 2009 ▶); cell refinement: *SAINT* (Bruker, 2009 ▶); data reduction: *SAINT*; program(s) used to solve structure: *SHELXS97* (Sheldrick, 2008 ▶); program(s) used to refine structure: *SHELXL97* (Sheldrick, 2008 ▶); molecular graphics: *ORTEP-3 for Windows* (Farrugia, 2012 ▶); software used to prepare material for publication: *PLATON* (Spek, 2009 ▶) and *publCIF* (Westrip, 2010 ▶).

## Supplementary Material

Crystal structure: contains datablock(s) I. DOI: 10.1107/S1600536814009283/rz5122sup1.cif

Structure factors: contains datablock(s) I. DOI: 10.1107/S1600536814009283/rz5122Isup2.hkl

Supporting information file. DOI: 10.1107/S1600536814009283/rz5122Isup3.cml

CCDC reference: 999285

Additional supporting information:  crystallographic information; 3D view; checkCIF report

## Figures and Tables

**Table 1:** Hydrogen-bond geometry (Å, °)

*D*—H⋯*A*	*D*—H	H⋯*A*	*D*⋯*A*	*D*—H⋯*A*
N3—H3*N*⋯O3^i^	0.84	2.14	2.960 (2)	164
C17—H17⋯O2^ii^	0.93	2.54	3.333 (3)	144

Symmetry codes: (i) 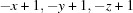; (ii) 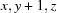.

**Fig. 1.**
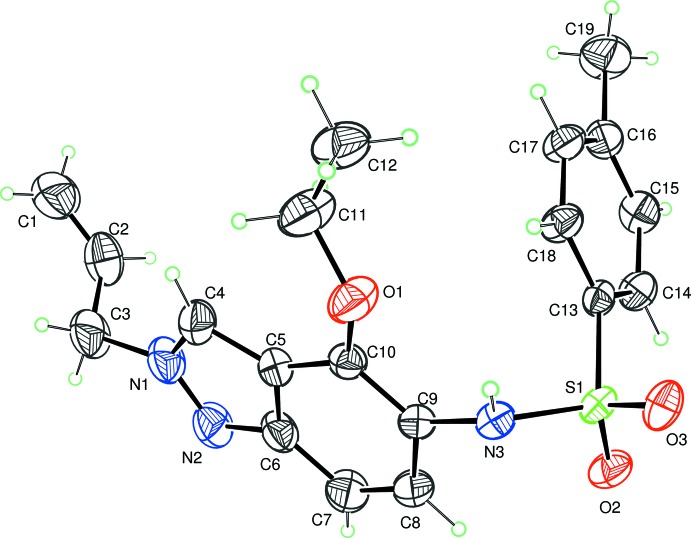
The molecular structure of the title compound with displacement ellipsoids drawn at the 50% probability level. H atoms are represented as small circles.

**Fig. 2.**
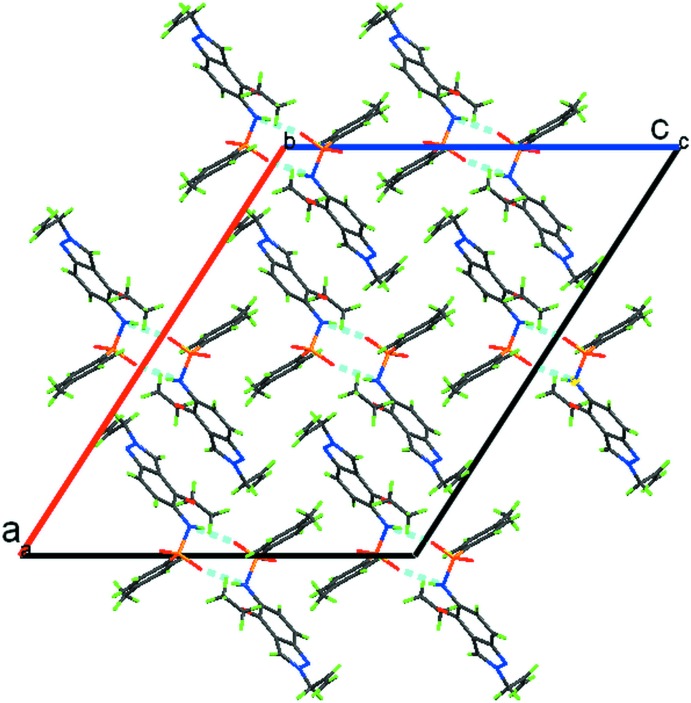
Projection of the crystal structure of the title compound along the b axis, showing molecules linked by hydrogen bonds (dashed lines).

## supplementary crystallographic information

### 1. Comment

Sulfonamides possess many types of biological activities and representatives of this class of pharmacological agents are widely used in clinic as antibacterial, hypoglycemic, diuretic and anti-carbonic anhydrase agents (Drews, 2000; Supuran & Scozzafava, 2001). Previously, a host of structurally novel sulfonamide derivatives have been reported to show substantial antitumor activity *in vitro* and/or *in vivo* (Abbate *et al.*, 2004; Rostom, 2006; Ghorab *et al.*, 2009). Recently, some *N*-[7(6)-indazolyl]arylsulfonamides prepared by our research group showed important antiproliferative activity against some human and murine cell lines ((Abbassi *et al.*, 2012; Abbassi *et al.*, 2013; Bouissane *et al.*, 2006).

The molecule of the title compound is built up from two fused almost coplanar five- and six-membered rings (N1/N2/C4-C10), with a maximum deviation of 0.029 (3) Å for atom C9 (Fig. 1). The indazole ring system is nearly perpendicular to the planes through the allyl group (C1–C3) and benzene ring (C13–C18) as indicated by the dihedral angles between them of 83.9 (3) and 77.99 (15)°, respectively. An intramolecular C—H···O hydrogen bond (Table 1) stabilizes the molecular comformation. The cohesion of the crystal structure is ensured by N3–H3N···O3 hydrogen bonds between centrosymmetrically related molecules forming dimers, which are further connected into columns parallel to the *b* axis by C17–H17···O2 hydrogen bonds (Fig. 2, Table 1).

### 2. Experimental

A mixture of 2-allyl-5-nitroindazole (1.22 mmol) and anhydrous SnCl_2_ (1.1 g, 6.1 mmol) in 25 ml of absolute ethanol was heated at 60°C for 6 h. After reduction, the starting material disappeared, and the solution was allowed to cool down. The pH was made slightly basic (pH 7–8) by addition of 5% aqueous potassium bicarbonate before extraction with ethyl acetate. The organic phase was washed with brine and dried over magnesium sulfate. The solvent was removed to afford the amine, which was immediately dissolved in pyridine (5 ml) and then reacted with 4-methylbenzenesulfonyl chloride (1.25 mmol) at room temperature for 24 h. The reaction mixture was then concentrated *in vacuo* and the resulting residue was purified by flash chromatography (eluted with ethyl acetate:hexane 2:8 *v*/*v*). The title compound was recrystallized from ethanol (yield = 78%, m. p. = 388 K).

### 3. Refinement

H atoms were located in a difference Fourier map and treated as riding with C–H = 0.93-0.97 Å, N–H = 0.84 Å, and with *U*_iso_(H) = 1.2 *U*_eq_ (C, N) or 1.5 *U*_eq_ for methyl H atoms. Three outliers (2 0 0, -2 0 2, 1 1 1) were omitted in the last cycles of refinement.

### Crystal data

**Table:**

C_19_H_21_N_3_O_3_S	*F*(000) = 1568
*M**_r_* = 371.45	*D*_x_ = 1.342 Mg m^−^^3^
Monoclinic, *C*2/*c*	Melting point: 388 K
Hall symbol: -C 2yc	Mo *K*α radiation, λ = 0.71073 Å
*a* = 26.0808 (5) Å	Cell parameters from 4059 reflections
*b* = 7.9335 (2) Å	θ = 2.3–27.1°
*c* = 21.1573 (4) Å	µ = 0.20 mm^−^^1^
β = 122.839 (1)°	*T* = 296 K
*V* = 3678.13 (14) Å^3^	Block, colourless
*Z* = 8	0.42 × 0.35 × 0.30 mm

### Data collection

**Table:**

Bruker X8 APEX diffractometer	4059 independent reflections
Radiation source: fine-focus sealed tube	3100 reflections with *I* > 2σ(*I*)
Graphite monochromator	*R*_int_ = 0.048
φ and ω scans	θ_max_ = 27.1°, θ_min_ = 2.3°
Absorption correction: multi-scan (*SADABS*; Bruker, 2009)	*h* = −33→33
*T*_min_ = 0.693, *T*_max_ = 0.747	*k* = −10→10
37135 measured reflections	*l* = −27→27

### Refinement

**Table:**

Refinement on *F*^2^	Primary atom site location: structure-invariant direct methods
Least-squares matrix: full	Secondary atom site location: difference Fourier map
*R*[*F*^2^ > 2σ(*F*^2^)] = 0.046	Hydrogen site location: inferred from neighbouring sites
*wR*(*F*^2^) = 0.134	H-atom parameters constrained
*S* = 1.07	*w* = 1/[σ^2^(*F*_o_^2^) + (0.0616*P*)^2^ + 3.2227*P*] where *P* = (*F*_o_^2^ + 2*F*_c_^2^)/3
4059 reflections	(Δ/σ)_max_ < 0.001
235 parameters	Δρ_max_ = 0.28 e Å^−^^3^
0 restraints	Δρ_min_ = −0.36 e Å^−^^3^

### Special details

**Table:**

Geometry. All e.s.d.'s (except the e.s.d. in the dihedral angle between two l.s. planes) are estimated using the full covariance matrix. The cell e.s.d.'s are taken into account individually in the estimation of e.s.d.'s in distances, angles and torsion angles; correlations between e.s.d.'s in cell parameters are only used when they are defined by crystal symmetry. An approximate (isotropic) treatment of cell e.s.d.'s is used for estimating e.s.d.'s involving l.s. planes.
Refinement. Refinement of *F*^2^ against all reflections. The weighted *R*-factor *wR* and goodness of fit *S* are based on *F*^2^, conventional *R*-factors *R* are based on *F*, with *F* set to zero for negative *F*^2^. The threshold expression of *F*^2^ > 2σ(*F*^2^) is used only for calculating *R*-factors(gt) *etc*. and is not relevant to the choice of reflections for refinement. *R*-factors based on *F*^2^ are statistically about twice as large as those based on *F*, and *R*- factors based on all data will be even larger.

### Fractional atomic coordinates and isotropic or equivalent isotropic displacement parameters (Å^2^)

**Table:**

	*x*	*y*	*z*	*U*_iso_*/*U*_eq_	
C1	0.18654 (15)	1.1635 (5)	0.01834 (19)	0.0893 (10)	
H1A	0.1582	1.1968	0.0297	0.107*	
H1B	0.2038	1.2431	0.0030	0.107*	
C2	0.20182 (12)	1.0079 (4)	0.02385 (15)	0.0683 (8)	
H2	0.2302	0.9786	0.0121	0.082*	
C3	0.17730 (12)	0.8734 (4)	0.04754 (16)	0.0724 (8)	
H3A	0.1511	0.8016	0.0047	0.087*	
H3B	0.1524	0.9234	0.0638	0.087*	
C4	0.26062 (10)	0.8146 (3)	0.18156 (14)	0.0550 (6)	
H4	0.2592	0.9161	0.2025	0.066*	
C5	0.30029 (9)	0.6795 (3)	0.21932 (12)	0.0414 (5)	
C6	0.28267 (10)	0.5576 (3)	0.16164 (13)	0.0483 (5)	
C7	0.31134 (11)	0.3998 (3)	0.17722 (15)	0.0596 (7)	
H7	0.2990	0.3198	0.1395	0.071*	
C8	0.35751 (10)	0.3678 (3)	0.24888 (13)	0.0496 (6)	
H9	0.3762	0.2625	0.2606	0.060*	
C9	0.37814 (9)	0.4902 (2)	0.30660 (11)	0.0356 (4)	
C10	0.35000 (9)	0.6438 (3)	0.29332 (11)	0.0363 (4)	
C11	0.36304 (15)	0.9237 (3)	0.34341 (16)	0.0712 (8)	
H11B	0.3205	0.9536	0.3207	0.085*	
H11A	0.3758	0.9615	0.3104	0.085*	
C12	0.40124 (14)	1.0063 (4)	0.41880 (16)	0.0715 (8)	
H12B	0.3969	1.1264	0.4130	0.107*	
H12A	0.4433	0.9761	0.4409	0.107*	
H12C	0.3880	0.9692	0.4509	0.107*	
C13	0.52408 (8)	0.5771 (2)	0.37746 (10)	0.0329 (4)	
C14	0.55443 (10)	0.5564 (3)	0.34089 (12)	0.0428 (5)	
H14	0.5586	0.4500	0.3258	0.051*	
C15	0.57834 (11)	0.6960 (3)	0.32728 (13)	0.0494 (6)	
H15	0.5995	0.6821	0.3036	0.059*	
C16	0.57191 (10)	0.8555 (3)	0.34757 (13)	0.0460 (5)	
C17	0.54115 (11)	0.8730 (3)	0.38424 (13)	0.0477 (5)	
H17	0.5363	0.9797	0.3984	0.057*	
C18	0.51797 (10)	0.7357 (3)	0.39973 (12)	0.0421 (5)	
H18	0.4982	0.7490	0.4251	0.050*	
C19	0.59695 (14)	1.0079 (4)	0.33072 (18)	0.0743 (8)	
H19A	0.6303	0.9746	0.3264	0.111*	
H19B	0.6110	1.0884	0.3707	0.111*	
H19C	0.5654	1.0579	0.2843	0.111*	
N1	0.22511 (9)	0.7686 (3)	0.10913 (12)	0.0592 (6)	
N2	0.23653 (9)	0.6139 (3)	0.09414 (12)	0.0616 (6)	
N3	0.42783 (7)	0.4479 (2)	0.38105 (9)	0.0368 (4)	
H3N	0.4322	0.5087	0.4163	0.044*	
O1	0.37042 (8)	0.7467 (2)	0.35341 (9)	0.0589 (5)	
O2	0.48852 (7)	0.26717 (19)	0.34847 (9)	0.0493 (4)	
O3	0.53203 (7)	0.3684 (2)	0.47767 (8)	0.0486 (4)	
S1	0.49532 (2)	0.39937 (6)	0.39815 (3)	0.03587 (16)	

### Atomic displacement parameters (Å^2^)

**Table:**

	*U*^11^	*U*^22^	*U*^33^	*U*^12^	*U*^13^	*U*^23^
C1	0.0645 (19)	0.080 (2)	0.091 (2)	−0.0064 (17)	0.0212 (18)	0.005 (2)
C2	0.0484 (14)	0.096 (2)	0.0516 (15)	0.0093 (15)	0.0215 (12)	0.0095 (16)
C3	0.0416 (13)	0.081 (2)	0.0604 (17)	0.0034 (13)	0.0054 (13)	0.0161 (15)
C4	0.0419 (12)	0.0544 (15)	0.0548 (15)	0.0074 (11)	0.0172 (11)	0.0020 (12)
C5	0.0333 (10)	0.0438 (12)	0.0446 (12)	0.0008 (9)	0.0195 (9)	0.0008 (10)
C6	0.0347 (10)	0.0561 (14)	0.0435 (12)	−0.0049 (10)	0.0144 (10)	−0.0076 (11)
C7	0.0518 (14)	0.0542 (15)	0.0531 (15)	−0.0049 (11)	0.0156 (12)	−0.0207 (12)
C8	0.0480 (12)	0.0370 (12)	0.0566 (14)	−0.0018 (9)	0.0236 (12)	−0.0097 (10)
C9	0.0352 (10)	0.0320 (10)	0.0392 (11)	−0.0016 (8)	0.0200 (9)	0.0006 (9)
C10	0.0370 (10)	0.0355 (11)	0.0380 (11)	−0.0032 (8)	0.0213 (9)	−0.0040 (9)
C11	0.094 (2)	0.0451 (16)	0.0664 (18)	0.0085 (14)	0.0381 (17)	−0.0006 (13)
C12	0.085 (2)	0.0484 (16)	0.080 (2)	−0.0012 (14)	0.0440 (17)	−0.0163 (14)
C13	0.0330 (9)	0.0321 (10)	0.0300 (9)	0.0052 (8)	0.0147 (8)	0.0033 (8)
C14	0.0478 (12)	0.0407 (12)	0.0449 (12)	0.0063 (9)	0.0284 (10)	−0.0007 (10)
C15	0.0514 (13)	0.0562 (15)	0.0532 (13)	0.0016 (11)	0.0365 (12)	0.0031 (11)
C16	0.0424 (11)	0.0458 (13)	0.0473 (13)	−0.0005 (10)	0.0227 (10)	0.0087 (10)
C17	0.0558 (13)	0.0327 (12)	0.0596 (14)	0.0042 (10)	0.0346 (12)	0.0016 (10)
C18	0.0509 (12)	0.0338 (11)	0.0524 (13)	0.0047 (9)	0.0352 (11)	0.0014 (10)
C19	0.0806 (19)	0.0632 (19)	0.095 (2)	−0.0091 (15)	0.0580 (18)	0.0138 (16)
N1	0.0365 (10)	0.0692 (15)	0.0498 (12)	0.0019 (9)	0.0089 (9)	0.0063 (11)
N2	0.0434 (11)	0.0702 (15)	0.0492 (12)	−0.0022 (10)	0.0109 (10)	−0.0057 (11)
N3	0.0419 (9)	0.0324 (9)	0.0389 (9)	0.0019 (7)	0.0238 (8)	0.0019 (7)
O1	0.0722 (11)	0.0430 (9)	0.0508 (10)	0.0097 (8)	0.0264 (9)	−0.0026 (8)
O2	0.0595 (10)	0.0317 (8)	0.0595 (10)	0.0054 (7)	0.0341 (8)	−0.0047 (7)
O3	0.0523 (9)	0.0491 (9)	0.0398 (8)	0.0167 (7)	0.0221 (7)	0.0171 (7)
S1	0.0418 (3)	0.0277 (3)	0.0375 (3)	0.0078 (2)	0.0211 (2)	0.0056 (2)

### Geometric parameters (Å, º)

**Table:**

C1—C2	1.284 (4)	C11—H11A	0.9700
C1—H1A	0.9300	C12—H12B	0.9600
C1—H1B	0.9300	C12—H12A	0.9600
C2—C3	1.465 (4)	C12—H12C	0.9600
C2—H2	0.9300	C13—C18	1.382 (3)
C3—N1	1.477 (3)	C13—C14	1.384 (3)
C3—H3A	0.9700	C13—S1	1.760 (2)
C3—H3B	0.9700	C14—C15	1.376 (3)
C4—N1	1.342 (3)	C14—H14	0.9300
C4—C5	1.400 (3)	C15—C16	1.375 (3)
C4—H4	0.9300	C15—H15	0.9300
C5—C10	1.418 (3)	C16—C17	1.393 (3)
C5—C6	1.424 (3)	C16—C19	1.506 (3)
C6—N2	1.350 (3)	C17—C18	1.369 (3)
C6—C7	1.402 (3)	C17—H17	0.9300
C7—C8	1.354 (3)	C18—H18	0.9300
C7—H7	0.9300	C19—H19A	0.9600
C8—C9	1.418 (3)	C19—H19B	0.9600
C8—H9	0.9300	C19—H19C	0.9600
C9—C10	1.370 (3)	N1—N2	1.341 (3)
C9—N3	1.435 (3)	N3—S1	1.6389 (16)
C10—O1	1.354 (3)	N3—H3N	0.8417
C11—O1	1.418 (3)	O2—S1	1.4261 (15)
C11—C12	1.497 (4)	O3—S1	1.4357 (15)
C11—H11B	0.9700		
			
C2—C1—H1A	120.0	C11—C12—H12C	109.5
C2—C1—H1B	120.0	H12B—C12—H12C	109.5
H1A—C1—H1B	120.0	H12A—C12—H12C	109.5
C1—C2—C3	124.1 (3)	C18—C13—C14	120.33 (19)
C1—C2—H2	118.0	C18—C13—S1	120.05 (15)
C3—C2—H2	118.0	C14—C13—S1	119.59 (16)
C2—C3—N1	113.3 (2)	C15—C14—C13	118.9 (2)
C2—C3—H3A	108.9	C15—C14—H14	120.6
N1—C3—H3A	108.9	C13—C14—H14	120.6
C2—C3—H3B	108.9	C16—C15—C14	122.0 (2)
N1—C3—H3B	108.9	C16—C15—H15	119.0
H3A—C3—H3B	107.7	C14—C15—H15	119.0
N1—C4—C5	106.4 (2)	C15—C16—C17	118.1 (2)
N1—C4—H4	126.8	C15—C16—C19	121.5 (2)
C5—C4—H4	126.8	C17—C16—C19	120.4 (2)
C4—C5—C10	137.2 (2)	C18—C17—C16	121.1 (2)
C4—C5—C6	103.6 (2)	C18—C17—H17	119.5
C10—C5—C6	119.2 (2)	C16—C17—H17	119.5
N2—C6—C7	126.8 (2)	C17—C18—C13	119.67 (19)
N2—C6—C5	111.7 (2)	C17—C18—H18	120.2
C7—C6—C5	121.4 (2)	C13—C18—H18	120.2
C8—C7—C6	117.9 (2)	C16—C19—H19A	109.5
C8—C7—H7	121.0	C16—C19—H19B	109.5
C6—C7—H7	121.0	H19A—C19—H19B	109.5
C7—C8—C9	121.8 (2)	C16—C19—H19C	109.5
C7—C8—H9	119.1	H19A—C19—H19C	109.5
C9—C8—H9	119.1	H19B—C19—H19C	109.5
C10—C9—C8	121.43 (19)	N2—N1—C4	114.5 (2)
C10—C9—N3	119.84 (17)	N2—N1—C3	119.7 (2)
C8—C9—N3	118.68 (18)	C4—N1—C3	125.8 (2)
O1—C10—C9	116.71 (18)	N1—N2—C6	103.7 (2)
O1—C10—C5	125.09 (19)	C9—N3—S1	121.32 (13)
C9—C10—C5	118.13 (18)	C9—N3—H3N	116.5
O1—C11—C12	108.4 (2)	S1—N3—H3N	108.9
O1—C11—H11B	110.0	C10—O1—C11	120.3 (2)
C12—C11—H11B	110.0	O2—S1—O3	118.36 (10)
O1—C11—H11A	110.0	O2—S1—N3	108.69 (9)
C12—C11—H11A	110.0	O3—S1—N3	104.65 (9)
H11B—C11—H11A	108.4	O2—S1—C13	107.78 (9)
C11—C12—H12B	109.5	O3—S1—C13	108.99 (10)
C11—C12—H12A	109.5	N3—S1—C13	107.96 (9)
H12B—C12—H12A	109.5		

### Hydrogen-bond geometry (Å, º)

**Table:**

*D*—H···*A*	*D*—H	H···*A*	*D*···*A*	*D*—H···*A*
C8—H9···O2	0.93	2.48	2.991 (3)	115
N3—H3*N*···O3^i^	0.84	2.14	2.960 (2)	164
C17—H17···O2^ii^	0.93	2.54	3.333 (3)	144

Symmetry codes: (i) −*x*+1, −*y*+1, −*z*+1; (ii) *x*, *y*+1, *z*.
