# *N*-(2-Allyl-4-eth­oxy-2*H*-indazol-5-yl)-4-methyl­benzene­sulfonamide. Corrigendum

**DOI:** 10.1107/S1600536814013439

**Published:** 2014-06-14

**Authors:** Hakima Chicha, El Mostapha Rakib, Latifa Bouissane, Maurizio Viale, Mohamed Saadi, Lahcen El Ammari

**Affiliations:** a Laboratoire de Chimie Organique et Analytique, Université Sultan Moulay Slimane, Faculté des Sciences et Techniques, Béni-Mellal, BP 523, Morocco; b IRCCS, Azienda Ospedaliera Universitaria San Martino–IST Istituto Nazionale per la Ricerca sul Cancro, U.O.C. Bioterapie, L.go R. Benzi 10, 16132 Genova, Italy; c Laboratoire de Chimie du Solide Appliquée, Faculté des Sciences, Université Mohammed V-Agdal, Avenue Ibn Battouta, BP 1014, Rabat, Morocco

## Abstract

The affiliation address of one of the authors in the paper by Chicha *et al.* [*Acta Cryst.* (2014), E**70**, o624] is corrected.

## Abstract 2

Corrigendum to *Acta Cryst.* (2014), E**70**, o624.

In the paper by Chicha *et al.* (2014) ▶, the affiliation address of ‘Maurizio Viale’ was incorrect. The correct address is given above.
